# Clinical outcome of different embryo transfer strategies after late rescue ICSI procedure: a 10-year total fertilisation failure cohort study

**DOI:** 10.1186/s12884-023-05859-0

**Published:** 2023-07-31

**Authors:** Xiaxuan Zhu, Tian Tian, Dina Jiesisibieke, Shilin Fang, Nan Zhang, Jinxi Ma, Yuqi Xia, Ping Liu, Rong Li, Jie Qiao, Rui Yang

**Affiliations:** 1 Center for Reproductive Medicine, Department of Obstetrics and Gynecology, Peking University Third Hospital, No. 49 North Huayuan Road, Haidian District, Beijing, 100191 China; 2 National Clinical Research Center for Obstetrics and Gynecology, Beijing, 100191 China; 3 Key Laboratory of Assisted Reproduction (Peking University), Ministry of Education, Beijing, 100191 China; 4 Beijing Key Laboratory of Reproductive Endocrinology and Assisted Reproductive Technology, Beijing, 100191 China

**Keywords:** Rescue intracytoplasmic sperm injection, Total fertilisation failure, In vitro blastulation, Freeze-all embryo transfer, Blastocyst

## Abstract

**Background:**

Late rescue intracytoplasmic sperm injection (r-ICSI) has not been widely accepted as an alternative solution for unexpected total fertilisation failure (TFF) after in vitro fertilisation (IVF), due to the time-dependent in vitro deterioration of oocyte quality and endometrial growth not being synchronised with embryo development. This study aimed to evaluate the safety profile and effectiveness of freeze-all blastocyst transfer in combination with late r-ICSI.

**Methods:**

This was a retrospective cohort study carried out at the Reproductive Centre of Peking University Third Hospital, Beijing, China. All participants received treatment between 2009 and 2019. 2,270 patients in the aggregate encountered unexpected TFF during 149,054 cycles of IVF and adopted a late r-ICSI procedure. Among these patients, 263 women did not have cleavage-stage embryos available for evaluation. The remaining patients were grouped according to different embryo transfer (ET) strategies (926 women in Group 1 underwent fresh ET, 365 women in Group 2 underwent freeze-all ET, 716 women in Group 3 experienced blastulation failure). Patients received different ET strategies after r-ICSI, with the main outcome measures included live birth rate (LBR), cumulative live birth rate (cLBR), and conservative cLBR.

**Results:**

TFF occurred in 7.4% of all IVF cycles. Group 1 tended to be older at oocyte retrieval, with more infertile years, higher follicle-stimulating hormone (FSH) levels, higher gonadotropin consumption, and fewer oocytes retrieved. Group 2 exhibited considerably better LBRs following the first ET cycle (37.53% vs. 4.64%) and cLBRs (52.60% vs. 8.21%). After adjustment for covariates using binary logistic regression analyses, Group 2 still showed better obstetric performance in LBRs [OR:11.77, 95% CI (8.42–16.45)], cLBRs (OR:11.29, 95% CI (7.84–16.27)], and conservative cLBRs (OR:2.55, 95% CI (1.83–3.55)]. Additionally, the two groups showed similar miscarriage rates, whilst no new-borns with malformations or congenital diseases were reported.

**Conclusions:**

Freeze-all blastocyst stage ET serves as an optimal strategy to support late r-ICSI. However, for women with limited oocytes available for r-ICSI use, weighing the benefits against the costs of the procedure might be prudent before implementing in vitro blastulation.

**Supplementary Information:**

The online version contains supplementary material available at 10.1186/s12884-023-05859-0.

## Introduction

Intracytoplasmic sperm injection (ICSI) is widely opted for routine reproductive medicine, however, it is not currently encouraged for use in patients with unexplained infertility, or in the absence of male factor infertility. Previous studies have shown a lack clear of evidence to support ICSI being more beneficial than conventional in vitro fertilisation (IVF) [1, 2]. Furthermore, ICSI is expensive and has been also associated with a higher risk of chromosome abnormalities, epigenetic disorders, and growth retardation than conventional IVF, however, this data was inconclusive [3]. Therefore, many reproductive medicine centres do not have a precise, standard protocol of use for ICSI [4].

However, the abnormal failure of fertilisation in all oocytes has been observed in conventional insemination in approximately 5.4–10.6% of IVF cycles performed [5–7], referred to as total fertilisation failure (TFF). TFF can be both emotionally and financially frustrating for patients undergoing IVF, and thus, the utilisation of ICSI would be beneficial here, although its use continues to be debated [5, 8, 9].

Rescue ICSI (r-ICSI), also known as delayed ICSI, is a potential practical strategy that could salvage clinical outcomes following unexpected TFF, and consists of the administration of ICSI 4–24 h after conventional insemination for oocytes that show no signs of fertilisation. In males with normal semen parameters who achieved fertilisation with r-ICSI, bioinformatic analyses revealed altered sperm proteins. Those differentially expressed proteins may have induced the sealed sperm and increased the spontaneous acrosome reaction rate [10, 11]. The biological basis of this further corroborated the validity of r-ICSI. Due to the difficulty of organising early r-ICSI (4–8 h after conventional insemination) caused by time limitations [12], late r-ICSI performed on 1-day-old mature oocytes has been adopted in many IVF units [13].

Nonetheless, its widespread use has been hindered by its low success rate, which may be a result of the time-dependent in vitro deterioration of oocyte quality and endometrial growth being unsynchronised from embryo development [12]. Recent years have witnessed enhanced endometrial synchronisation after the employment of frozen-thawed embryo transfer (FET). Observational studies have revealed that embryos generated from late r-ICSI that were transferred after cryopreservation in a frozen-thawed cycle yielded superior results [14, 15]. Furthermore, blastocyst culture is an available and plausible strategy for reducing the prevalence of aneuploidy to counteract the effects of oocyte ageing in r-ICSI cycles and to therefore guarantee good development potential [16]. Current evidence shows the moderate superiority of blastocysts compared to cleavage-stage ET in live birth and clinical pregnancy outcomes and, more importantly, in improved transfer efficiency [17–19].

Large-scale research identifying the link between r-ICSI strategies and obstetric outcomes remains relatively scarce [13, 14]. Thus, here we aspired to corroborate whether r-ICSI alongside blastocyst cultures and frozen ET could improve the assisted reproductive technology (ART) success rate of couples experiencing TFF following conventional IVF cycles.

## Materials and methods

### Study population

Data of the participants using r-ICSI were extracted from a large dataset based on the medical records of couples who underwent ART procedures between 2009 and 2019 at the Centre for Reproductive Medicine, Peking University Third Hospital. As one of the largest reproductive health centres in China, this centre has recorded information on ovarian stimulation (OS) cycles, followed by 149,054 fresh embryo transfer (fresh ET) and 79,874 FET cycles. Notably, we excluded participants who accepted the donor sperm or implementation of pre-implantation genetic testing (PGT) procedures.

**Fig. 1:**
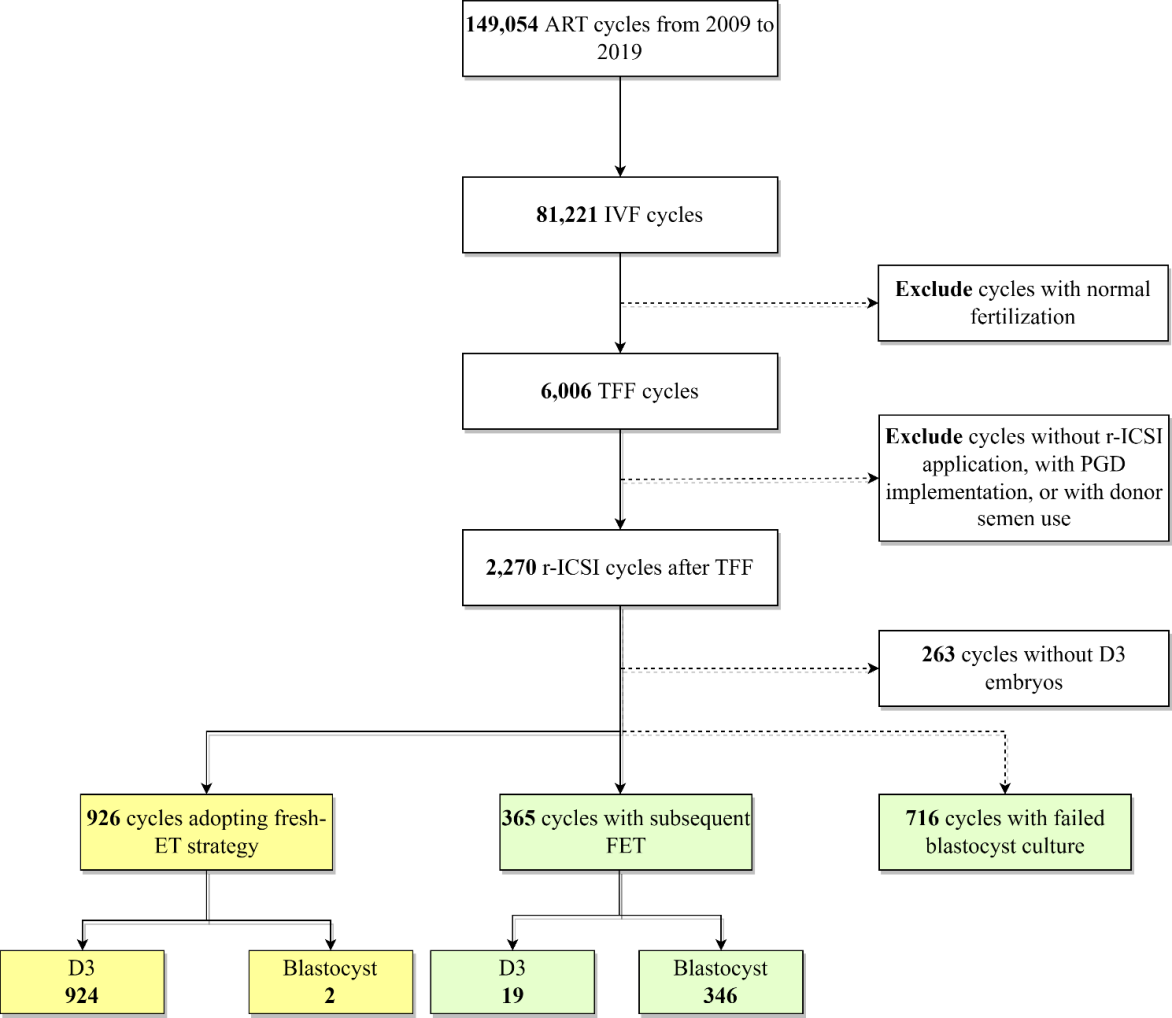
Flow chart showing selection and exclusions of women adopting freeze-all or fresh transfer strategy in r-ICSI cycles. *Note*: ART = assisted reproductive technology; r-ICSI = rescue intracytoplasmic sperm injection; TFF = total fertilization failure; FET = frozen embryo transfer; fresh-ET = fresh-embryo transfer

Out of all ART processes, r-ICSI cycles were included, whilst cycles mixed with embryos acquired through other insemination methods such as IVF and/or ICSI were excluded. Finally, 2,270 r-ICSI cycles encountered unexpected TFF across 149,054 cycles of IVF and subsequently adopted a late r-ICSI procedure. Of these patients, 263 women did not have cleavage-stage embryos available for evaluation. The remaining patients were categorized into three groups based on their different embryo transfer (ET) strategies. Specifically, Group 1 consisted of 926 women who underwent fresh ET; Group 2 included 365 women who underwent a freeze-all ET strategy, and Group 3 contained 716 women who experienced blastulation failure (Fig. 1).

We considered the following variables when regarding OS cycles: Essential characteristics of participants [age, infertility years, body mass index (BMI), etc.], causes of infertility [female and male], the level of based serum reproductive hormone [follicle-stimulating hormone (FSH), oestradiol (E2), luteinising hormone (LH), and prolactin (PRL)], medication use, and other variables related to the treatment process.

### Ethical considerations

This study has obtained approval from the Ethics Committee of the Peking University Third Hospital for data handling (No. IRB00006761-M2020007). All procedures conformed to the ethical standards of the responsible committee for human experimentation, and the Helsinki Declaration of 1964 and its later amendments. All patients included in this study gave informed consent.

### Laboratory procedures

OS was mainly performed using either a short antagonist protocol, or long agonist protocol coupled with recombinant FSH (Gonal-F alfa, Merck Serono, Germany), recombinant follitropin beta (Puregon, MSD, USA), urofollitropin (Livzon Pharmaceutical Group Inc., China), or menotrophins (Livzon Pharmaceutical Group Inc., China) administered on days 2 or 3 of the menstrual cycle. When the follicles reached 10–12 mm, a GnRH antagonist (Cetrotide, Merck Serono, Germany; Ganirelix, MSD, USA) was applied. Otherwise, GnRH agonists (Triptorelin, Ipsen Pharma Biotech, France) were administered in the last follicle or luteal phase in the ultra-long and long protocols, or one day before gonadotrophin application in the short protocol. When no less than two dominant follicles measuring > 18 mm had been testified by ultrasound, final maturation and ovulation were triggered with human chorionic gonadotropin (hCG, 250 µg) (Choriogonadotropin alfa, Merck Serono, Germany) administration. On other occasions, hCG (1000-2000IU of 250 ug) and GnRH agonist (0.2 mg) were administered in dual trigger cycles, with 0.2 mg GnRH agonist being used as a trigger, according to clinical conditions for high response cycles in the antagonist protocol. Oocyte retrieval was subsequently performed after 36–38 h.

In our Centre for Reproductive Medicine, ICSI is incorporated as a standard procedure under specific conditions including severe male factor infertility, prior fertilization failure. In absence of these, conventional IVF is generally utilized.

The semen analyses performed were in accordance with the World Health Organization (WHO) guidelines. Specifically, we used the criteria outlined in the 4th and 5th editions of the WHO Laboratory Manual for the Examination and Processing of Human Semen. Normozoospermic samples were selected by analysis of semen concentration, motility, and morphology. Oocytes were then examined for the existence of a second polar body after exposure to spermatozoa for 16–19 h. Those without an evident second polar body in all oocytes after IVF insemination, also referred to as TFF, were subjected to r-ICSI within one hour using 1-day semen sample, which was performed in accordance with the standard ICSI procedure [20] And subsequently checked for fertilisation the next day. The two-pronuclear (2PN) fertilisation rate was calculated as the number of oocytes with two pronuclei acquired using the r-ICSI method/number of all retrieved oocytes. One to three embryos were then transferred from the same fresh cycle, accompanied by luteal phase progesterone support (Progesterone Vaginal Gel, Merck Serono, Germany). After this, embryos were cryopreserved using vitrification technology for future FET, carried out in natural or hormone-artificial cycles according to routine clinical procedures, which have been detailed elsewhere [21]. In the case of fresh r-ICSI cycles, cleavage-stage embryos were transferred on Day 3 corresponding to the embryo’s development stage of Day 2. For blastocysts in fresh cycles, the transfer was performed on Day 6 reflecting the blastocyst’s development stage of Day 5. In FET cycles, the transfers were conducted conventionally on either Day 3 or Day 5, depending on the embryo’s developmental stage.

### Study outcomes

Crucial indicators for outcomes followed the Chinese Society of Reproductive Medicine (CSRM) consensus for consistency in clinical operations [22]. A positive hCG result was determined through blood β-hCG testing 12 to 14 days post-embryo transfer, with a threshold level established at greater than 30 mIU/ml to signify rising hCG. Women who tested positive received an ultrasound examination at least 28–30 days after transfer to detect the existence of a gestational sac, which was then confirmed as a clinical pregnancy. The embryo implantation rate (IR) was subsequently calculated as the number of gestational sacs/the number of embryos transferred. Miscarriage was defined as pregnancy loss before 28 weeks, and the miscarriage rate (MR) was also calculated for pregnant women. The primary objectives for the study were the delivery of a living foetus (or living foetuses) during the first cycle and all cycles after one OS procedure, which were separately associated with the live birth rate (LBR) and cumulative live birth rate (cLBR) [23]. The cLBR follow-up period ended in December 2019.

### Sensitivity analysis

To test the robustness of this study, we performed several sensitivity analyses. Firstly, to minimise the potential confounding effects of maternal age, we performed stratified analysis by dividing the population into two subgroups based on maternal age (< 35 or ≥ 35 years) before testing the associations between ET strategies and IVF outcomes for the two respective subgroups. We also performed propensity score matching (PSM) [24] to generate matched pairs in a 1:1 ratio balanced with age.

In addition, among our participants, 716 cycles adopting the blastocyst culture strategy in the stimulation cycle suffered from embryo loss and failed. Frozen blastocyst transfer has been reported to result in a higher LBR [17] and has also been adopted as a routine practice in the centre. Therefore, we performed an intention-to-treat (ITT) analysis assuming that all 716 cycles would choose to perform the FET strategy if embryos were available, and then included these in the Full Analysis Set (FAS), from which conservative cLBR was calculated [25].

### Statistical analyses

Baseline characteristics, including demographic variables, hormone parameters, and obstetric outcomes, were compared among the groups. In this study, considering that most of the continuous variables were not normally distributed, these were represented as medians and interquartile ranges [IQRs] and subsequently compared using the Mann–Whitney U test. Categorical variables were described as numbers or percentages and then compared using Pearson’s chi-square test. Binary logistic regression was also used to adjust for potential maternal confounding factors, which appeared to be statistically significant in the univariate analysis or were deemed critical. Additionally, it should be noted that the gonadotropin dose was log-transformed before the adjustment. All statistical analyses were conducted using IBM SPSS 26.0 (IBM Corp. Armonk, NY), with two-tailed tests being performed, and statistical significance set at p < 0.05.

## Results

### Characteristics of patients in the OS cycle

After routine IVF procedures, 7.4% (6006/81,221) of the individuals experienced TFF. A total of 2,270 OS cycles adopting the r-ICSI strategy were included among the women aged between 20 and 50 years who received treatment from January 2009 to December 2019. Of all individuals, 40.8% (926/2270, Group 1) of patients opted for the fresh-ET strategy, whilst 16.1% (365/2270, Group 2) selected the freeze-all ET strategy. Notably, for up to 43.1% of women (979/2270), researchers failed to obtain embryos eligible for transfer after oocyte retrieval, among which 73.4% of women (716/979, Group 3) suffered from transfer cancellation during blastocyst culturing (Fig. 1). Furthermore, the proportion of women without eligible embryos among all r-ICSI patients attributable to blastocyst culturing was 49.4%.

The baseline characteristics of the women during OS cycles are displayed in Table 1. Comparisons between Group 1 and Group 2 revealed significant differences, including maternal age [33.00 (30.00–37.00) vs. 31.00 (29.00–35.00), P < 0.001], infertile years [5.00 (3.00–8.00) vs. 3.00 (2.00–6.00), P < 0.001], levels of FSH [7.10 (5.71–8.69) vs. 6.61 (5.25–7.95) mIU/mL, P < 0.001], and dose of gonadotropins [2850.00 (1950.00–4125.00) vs. 2325.00 (1800.00–3150.00) IU, P < 0.001]. Moreover, Group 1 had a higher tendency towards secondary infertility. Specifically, a higher proportion of women in Group 1 had experienced one (20.73 vs. 19.18%), two (12.63 vs.6.03%), or even more than three pregnancies (9.72 vs. 3.56%) compared with Group 2. Women undergoing fresh ET were exhibited lower oocyte retrieval after stimulation [10.00 (6.00–15.00) vs. 13.00 (9.00–19.00), P < 0.001]; thus, fewer oocytes were retrieved for r-ICSI use [8.00 (5.00–12.00) vs. 10.00 (7.00–14.00), P < 0.001]. Additional comparisons were performed between groups 2 and 3 (Table S4). The latter turned out to be older [33.00 (30.00–37.00) vs. 31.00 (29.00–34.00), P < 0.001], with lower levels of LH [3.07 (1.81–4.51) vs. 3.52 (2.08–5.05), P = 0.008], received higher doses of gonadotropin [2775.00 (1881.25–3750.00) vs. 2250.00 (1650.00–3150.00), P < 0.001], and had lower oocyte retrieval after stimulation [9.00 (7.00–13.00) vs. 13.00 (9.00–18.00), P < 0.001]. The rates of 2PN fertilization following r-ICSI were recorded as 0.50 (0.25–0.71) in Group 1 and 0.54 (0.33–0.72) in Group 2, respectively. Moreover, the number of cleavage-stage embryos showed significant differences between the two groups [6.00 (3.75–9.00) vs. 8.00 (6.00–11.00), P < 0.001].

**Table 1:** Baseline characteristics and clinical outcomes of women undergoing fresh or freeze-all strategy in r-ICSI cycles

	Group 1	Group 2	P
	Fresh embryo transfer (N = 926)	Freeze-all-embryos (N = 365)	
**Maternal age at oocyte retrieval (IQR) — yr**	33.00 (30.00–37.00)	31.00 (29.00–35.00)	< 0.001
**Maternal BMI (IQR) —kg·m** ^**2**^	22.32 (20.20-24.65)	21.88 (20.20-24.01)	0.260
**Infertile years (IQR) — yr**	5.00 (3.00–8.00)	3.00 (2.00–6.00)	< 0.001
**Gravidity—no./total no. (%)**			< 0.001
**0**	562/926 (60.69)	260/365 (71.23)	
**1**	192/926 (20.73)	70/365 (19.18)	
**2**	117/926 (12.63)	22/365 (6.03)	
**≥ 3**	90/926 (9.72)	13/365 (3.56)	
**FSH(IQR) —mIU/mL**	7.10 (5.71–8.69)	6.61 (5.25–7.95)	< 0.001
**E2(IQR) —pg/mL**	148.00 (113.50-197.50)	159.00 (121.00-210.00)	0.766
**PRL(IQR) —nmol/L**	12.40 (8.88–17.60)	12.03 (9.16–16.70)	0.802
**LH(IQR) —mIU/mL**	3.50 (2.48–5.01)	3.90 (2.65–5.29)	0.733
**Gonadotropin dose(IQR)—IU**	2850.00 (1950.00-4125.00)	2325.00 (1800.00-3150.00)	< 0.001
**Lg (Gn)**	3.35 (3.21–3.49)	3.45 (3.29–3.60)	< 0.001
**Number of oocytes retrieval(IQR)**	10.00 (6.00–15.00)	13.00 (9.00–19.00)	< 0.001
**Number of oocytes used for r-ICSI**	8.00 (5.00–12.00)	10.00 (7.00–14.00)	< 0.001
**r-ICSI 2PN fertilization**	0.50 (0.25–0.71)	0.54 (0.33–0.72)	0.028
**r-ICSI cleavage embryos**	6.00 (3.75-9.00)	8.00 (6.00–11.00)	< 0.001
**positive hCG—no./total no. (%)**	101/926 (10.91)	200/365 (54.79)	< 0.001
**Clinical pregnancy—no./total no. (%)**	61/926 (6.59)	173/365 (47.39)	< 0.001
**IR—no./total no. (%)**	76/1971 (3.86)	184/433 (42.49)	< 0.001
**MR—no./total no. (%)**	18/61 (29.51)	36/173 (20.81)	0.166
**LBR—no./total no. (%)**	43/926(4.64)	137/365 (37.53)	< 0.001
**Newborns (N)**	50	146	-
**Cumulative clinical pregnancy—no./total no. (%)**	76/926 (8.21)	192/365 (52.60)	< 0.001
**cLBR—no./total no. (%)**	57/926 (6.16)	158/365 (43.29)	< 0.001

Note: IR = implantation rate; MR = miscarriage rate; LBR = live birth rate; cLBR = cumulative live birth rate

### Clinical outcomes of the first embryo transfer cycle and cumulative live birth rates

**Fig. 2:**
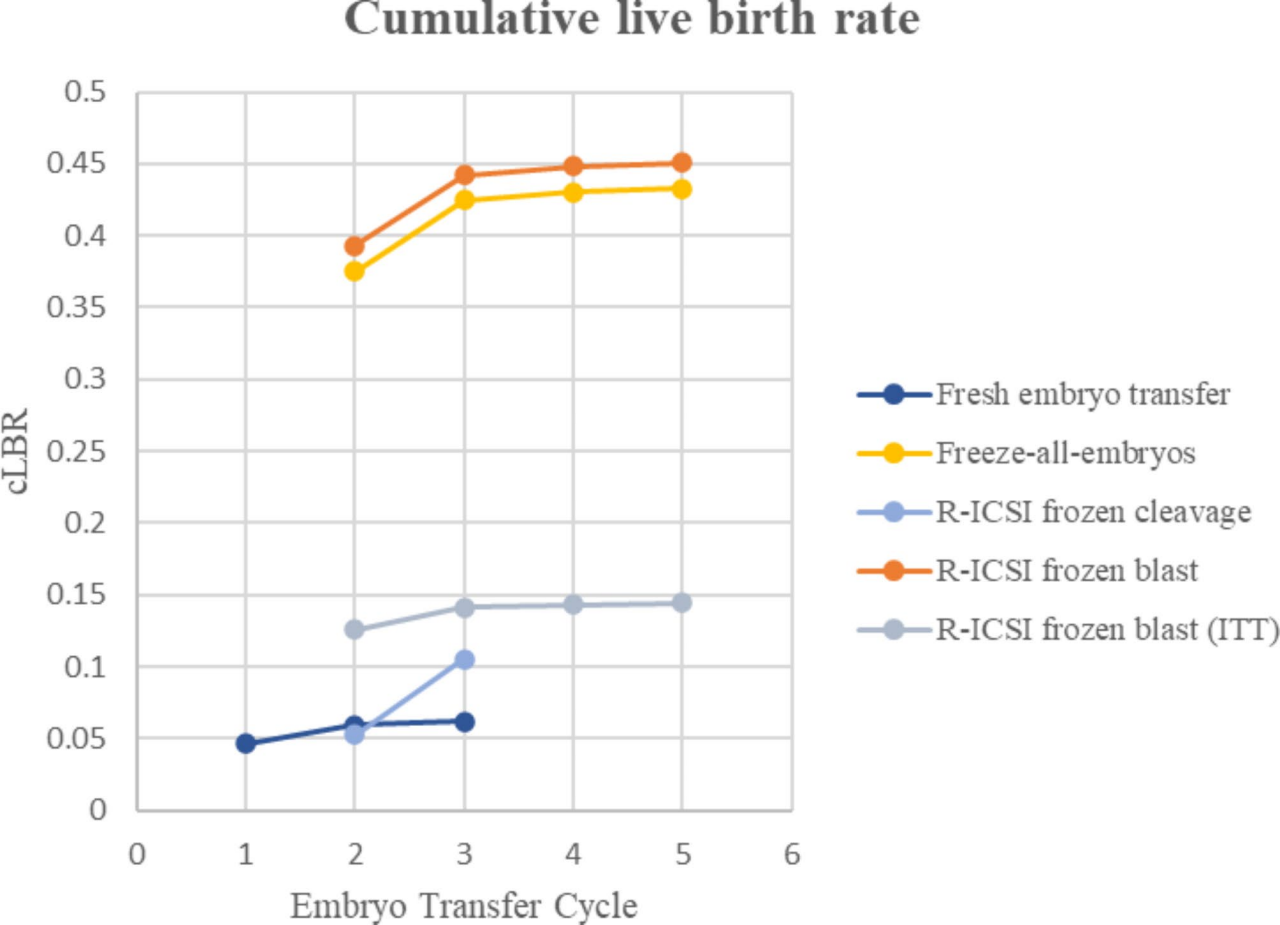
Cumulative live birth rate of women undergoing fresh or freeze-all strategy in r-ICSI cycles. *Note*: R-ICSI = rescue intracytoplasmic sperm injection; ITT = intention-to-treat.

Compared with women who underwent fresh ET in the stimulation cycle, a higher proportion of individuals within the freeze-all-embryo group exhibited a positive hCG rate (54.79 vs. 10.91%) and positive ultrasound examination results (47.39 vs. 6.59%), higher IR (42.49 vs. 3.86%), and similar MR (19.65 vs. 27.87%); thus, achieving considerably better clinical outcomes in both the LBR of the first ET cycle (37.53 vs. 4.64%) and cLBR (52.60 vs. 8.21%) (Table 1). Notably, women in Group 2 showed a remarkable improvement in LBR compared to others with fresh ET, whilst having more embryos available for extra transfer cycles (Fig. 2). Furthermore, no malformations were observed among the newborns.

### Associations between ET strategies and obstetric outcomes

Further inspection of the data showed that the fresh ET group had a more advanced maternal age (38.00 vs. 37.00) in the 35 years and up cohort, even after stratification of analysis according to maternal age. Women under 35 years of age had higher clinical pregnancy rates (8.14 vs. 4.29% in the fresh ET group and 48.96 vs. 41.55% in the freeze-all group), LBR (5.97 vs. 2.68% in the fresh ET group and 38.89 vs. 32.47% in the freeze-all group), and cLBR (10.13 vs. 5.36% in the fresh ET group and 53.82 vs. 48.05% in the freeze-all group), along with fewer infertile years, lower FSH levels, lower gonadotropin dose, and fewer oocytes used for r-ICSI. Although the freeze-all group stood out for both the younger and older cohorts, age stratification seemingly failed to mitigate the effects of confounding variables (Table S1). Among the 450 women who were 35 years or older, the eldest participant was 46 years old. A further stratification between women aged ≥ 35 and < 40 years and those aged ≥ 40 and ≤ 46 years revealed consistent trends in LBR and cLBR favouring the freeze-all ET group (Table S5).

Another approach involved producing an equal number of cohorts from the fresh ET and freeze-all groups by propensity score matching. While the fresh ET group was still inclined to have more infertile years [5.00 (3.00–7.00) vs. 4.00 (2.00–6.00), P < 0.001] and higher FSH levels [7.00 (5.58–8.34) vs. 6.09 (4.63–7.71), P < 0.001], there were no marked differences in oocytes remained for r-ICSI [10.00 (6.00–14.00) vs. 10.00 (7.00–14.00), P = 0.149]. Despite this, women who received fresh ET and freeze-all ET demonstrated significant differences in positive hCG rate (31.36 vs. 54.94%), clinical pregnancy (16.76 vs. 47.53%), IR (9.93 vs. 42.59%), LBR (11.81 vs. 37.64%), and cLBR (11.81 vs. 43.96%) (P < 0.001) (Table S2).

To confirm the efficacy of freeze-all ET, the most conserved estimates obtained using the ITT method were applied. As shown in Table S3, gaps between the two groups in the conservative cumulative clinical pregnancy (8.21 vs. 17.76%) and cLBR (6.16 vs. 14.62%) shrunk, although remained significant (P < 0.001).

After adjusting for covariates, including age, BMI, years of infertility, gravity, LH and FSH levels, Gn dose, number of oocytes, and 2PN fertilisation rate, using binary logistic regression analysis, the r-ICSI freeze-all ET method showed a higher likelihood of better obstetric outcomes, compared with those of the fresh ET method for LBR [OR:11.77, 95% CI (8.42–16.45)], cLBR [OR:11.29, 95% CI (7.84–16.27)] and conservative cLBR [OR:2.55, 95% CI (1.83–3.55)] (Table 2).

**Table 2:** Binary logistic regression analysis of factors associated with LBR, cLBR, and conservative cLBR.

	LBR OR (95%CI)	P	cLBR OR (95%CI)	P	Conservative cLBR OR (95%CI)	P
**Maternal age at oocyte retrieval (IQR) — yr**	0.94 (0.89–1.01)	0.082	0.97 (0.91–1.07)	0.315	0.95 (0.91-1.00)	0.030
**Maternal BMI (IQR) —kg·m** ^**2**^	0.94 (0.860–1.02)	0.09	0.94 (0.87–1.01)	0.106	0.96 (0.91-1.00)	0.063
**Infertile years (IQR) — yr**	1.02 (0.94–1.09)	0.707	0.99 (0.92–1.07)	0.782	1.01 (0.95–1.05)	0.861
**Gravidity — no./total no. (%)**	0.88 (0.65–1.20)	0.430	0.86 (0.64–1.17)	0.335	0.81 (0.65–1.02)	0.073
**FSH(IQR) —mIU/mL**	1.06 (0.97–1.17)	0.202	1.03 (0.94–1.13)	0.482	0.99 (0.95–1.04)	0.778
**LH(IQR) —mIU/mL**	0.88 (0.80–0.98)	0.016	0.92 (0.84-1.00)	0.061	0.99 (0.93–1.04)	0.640
**Gonadotropin dose(IQR)—IU**	1.00 (1.00–1.00)	0.938	1.00 (1.00–1.00)	0.520	1.00 (1.00–1.00)	0.646
**Number of oocytes retrieval (IQR)**	1.05 (0.98–1.12)	0. 179	1.05 (0.99–1.13)	0.120	1.06 (1.03–1.08)	< 0.001
**r-ICSI 2PN fertilization**	1.76 (0.78–3.96)	0.172	2.55 (1.15–5.65)	0.021	2.09 (1.19–3.66)	0.011
**Method**						
**Fresh ET**	Reference		Reference		Reference	
**Freeze-all embryos and FET**	10.85 (6.56–17.93)	< 0.001	12.12 (7.48–19.62)	< 0.001	2.66 (1.87–3.78)	< 0.001

Note: ET = embryo transfer; FET = frozen embryo transfer; LBR = live birth rate; cLBR = cumulative live birth rate

## Discussion

In this study, a large sample size was used, consisting of 149,054 IVF/ICSI fresh cycles and 79,874 frozen-thawed cycles, in an attempt to provide new evidence to support the use of the r-ICSI procedure. Our results subsequently demonstrated that r-ICSI is a safe and effective alternative for frustrated patients who experienced unexpected TFF following conventional IVF. In comparison with fresh ET, we showed that freeze-all blastocyst ET was an ideal strategy, which provided better obstetric outcomes, after late r-ICSI. Notably, maternal age, as well as the number of oocytes used for r-ICSI, determined the success of blastocyst cultures to a great extent and should therefore be considered in clinical decision-making.

TFF following IVF has mainly been attributed to unexplained causes [26], making it unpredictable for physicians. In clinical embryology, mechanistic events following human IVF can be divided into pre-sperm penetration and post-sperm penetration [27]. When TFF occurs spontaneously, a considerable amount of the causes related to pre-sperm penetration can be remedied by treatments such as r-ICSI, since the oocyte membrane is mechanically pierced, and physical obstacles are surpassed [10, 12, 20]. Our results also verified the potential of delayed oocyte activation and embryo development, whilst displaying a satisfactory 2PN fertilisation rate (50% and 54% in fresh and freeze-all ET, respectively).

During conventional IVF, the prolonged co-culture of oocytes and sperm is intended to improve embryo morphology and decrease the polyspermy rate compared with short-term IVF groups [28, 29]. Following insemination defeat, late r-ICSI in current clinical routine practices faces enormous challenges, including oocyte ageing after a one-day fertilisation procedure delay, weak endurance for prolonged environmental exposure, and unsynchronised endometrial growth [12, 13]. In line with previous research [13, 14], the results of our research examined a significant association between ET strategy and obstetric outcomes in individuals with TFF. Early investigations showed that pregnancy rate and LBR obtained following the fresh ET strategy were unsatisfactory [30]. In comparison, freeze-all ET was found to have significantly elevated the clinical pregnancy rate, LBR, and cLBR. Furthermore, positive effects remained predominant even after multiple adjustments, including stratified analysis, PSM, and binary logistic regression, eliminating the impacts of other covariates. Additionally, the FAS of the freeze-all ET group included 716 women who experienced embryo loss. Hence, the conservative cLBR was calculated to assess its clinical efficacy, while freeze-all ET showed a statistically significant cLBR. Interestingly, in comparison to conventional IVF or ICSI procedures, the freeze-all ET strategy was not superior to the others in terms of ongoing pregnancy rate and cLBR [31, 32].

A major finding of our study was that the freeze-all strategy emerges as the optimal approach in facilitating late r-ICSI. Intriguingly, in the context of conventional IVF or ICSI procedures, the freeze-all ET strategy did not exhibit superiority over fresh transfer in terms of the ongoing pregnancy rate and cLBR [31, 32]. An interesting question raised by this conclusion is why vitrification contributes to better outcomes in r-ICSI cycles compared to fresh embryos. The synchronization between the embryo’s developmental stage and the endometrial receptivity, known as the ‘implantation window’, is critical for successful implantation and subsequent pregnancy. With fresh transfer cycles, the process of ovarian stimulation can potentially disrupt this synchronization, leading to a suboptimal implantation environment [33]. On the other hand, the freeze-all strategy allows for better planning and control of the transfer timing, enhancing the likelihood of transfer occurring during the optimal implantation window. From this perspective, obstetric outcomes greatly benefitted from vitrification technology when asynchronization was obvious.

This study also revealed that essential characteristics varied significantly between the freeze-all group and those that failed to blastulate. The latter group was more likely to be older at oocyte retrieval, with higher LH levels, greater gonadotropin consumption, and lower oocytes retrieval for r-ICSI, primarily due to the adverse maternal environment. Consequently, for women with fewer oocytes available for r-ICSI use, great caution should be exercised when implementing in vitro blastulation to prevent unnecessary costs, as failure is more likely to occur. Notably, previous research suggests that only a small fraction of patients truly benefit from extended embryo culture, further emphasizing the need for judicious clinical decision-making [34].

The findings based on this large dataset also validated the safety and feasibility of r-ICSI as an alternative method for individuals with TFF. Admittedly, the MR of late r-ICSI was nearly twice the average MR of conventional IVF/ICSI procedures (approximately 6.5–10.1%)[35, 36], and was also slightly higher than the previous statistics on late r-ICSI [13], which was partially due to the older participants enrolled in our analysis. It is also noteworthy that late r-ICSI had little association with increased clinical risk, since no new-borns with malformations or congenital diseases were reported.

Our study presents several strengths. Firstly, the original data were derived from a large and anonymised dataset collected from a reproductive health centre in China. Since only a portion of women who experienced TFF chose the r-ICSI procedure, our study, combined with detailed medical information, could provide strong evidence whilst supplementing previous findings. Due to rich diversity among infertile couples, we ensured the replicability of our findings in different clinical settings. Furthermore, we performed several sensitivity analyses to ensure the robustness of our results. For example, we conducted subgroup analysis stratified by maternal age, PSM to balance the comparability of two groups, and a binary regression model to determine the interference by covariates. In addition, we applied an FAS in the freeze-all ET group, including women who experienced blastocyst culture failure. cLBR was still significantly ameliorated in this subjectively enlarged group, compared to that in the fresh ET group.

One limitation of this study was its retrospective design, which was inevitably associated with a skewed data distribution and inherent biases. The imbalance between cleavage-stage embryos and blastocysts could potentially confound our results. To address this, we considered the cLBR, in line with previous studies showing no significant difference between cleavage and blastocyst stage embryos [13]. This approach allowed us to minimise the impact of embryonic factors and concentrate on the outcomes of our clinical strategy. It should be noted, however, that complete avoidance of confounding, especially considering the higher fertilization rates with blastocysts, is challenging. Future randomised controlled trials with long-term follow-up are indispensable for evaluating the safety and efficacy of r-ICSI using a freeze-all ET strategy.

## Conclusion

Overall, our research validated the safety and feasibility of r-ICSI in individuals who have experienced TFF. Moreover, the current study suggested that the most optimal strategy for r-ICSI was frozen-thawed blastocyst ET, since the cLBR was improved even with modest estimates, including blastocyst culture failure. Although the overall success rate of r-ICSI remained within the range of poor prognosis, investigations to improve decisions are still necessary to help reproductive specialists deal with patients experiencing TFF and to subsequently develop individualised treatment plans.

## Electronic supplementary material

Below is the link to the electronic supplementary material.

Supplementary Material 1

## Data Availability

Data regarding any of the subjects in the study has not been previously published unless specified. The datasets used and analysed during the current study available from the corresponding author on reasonable request.

## List of abbreviations

ICSI: Intracytoplasmic sperm injection
IVF: In vitro fertilisation
TFF: Total fertilisation failure
r-ICSI: Rescue intracytoplasmic sperm injection
FET: Frozen-thawed embryo transfer
ART: Assisted reproductive technology
OS: Ovarian stimulation
ET: Embryo transfer
BMI: Body mass index
FSH: Follicle-stimulating hormone
E2: Oestradiol
LH: Luteinising hormone
PRL: Prolactin
GnRH: Gonadotropin-releasing hormone
hCG: Human chorionic gonadotrophin
CSRM: Chinese Society of Reproductive Medicine
IR: Implantation rate
MR: Miscarriage rate
LBR: Live birth rate
cLBR: Cumulative live birth rate
PSM: Propensity score matching
ITT: Intention-to-treat
FAS: Full Analysis Set
IQRs: Interquartile ranges
OR: Odds ratio
CI: Confidence interval
