# Supplementary material for: Clinical outcome of different embryo transfer strategies after late rescue ICSI procedure: a 10-year total fertilisation failure cohort study
Source: BMC Pregnancy Childbirth. 2023 Jul 31;23:549. doi: 10.1186/s12884-023-05859-0 (PMC10388511; doi:10.1186/s12884-023-05859-0)
Supplement: Supplementary file 1 — Supplementary Material 1 [file 12884_2023_5859_MOESM1_ESM.docx]

# Supplemental table 1.

**Supplemental table 1.** Demographic characteristics and clinical outcomes of participants after age-stratified analysis.

|  | **<35 years (N=841)** | | | **≥35 years(N=450)** | | |
| --- | --- | --- | --- | --- | --- | --- |
|  | **Fresh embryo transfer**  **(N=553)** | **Freeze-all-embryos(N=288)** | **P** | **Fresh embryo transfer**  **(N=373)** | **Freeze-all-embryos(N=77)** | **P** |
| **Maternal age at oocyte retrieval (IQR) — yr** | 31.00 (29.00-33.00) | 30.00 (28.00-32.00) | 0.372 | 38.00 (36.00-40.00) | 37.00 (35.00-38.00) | 0.001 |
| **Maternal BMI (IQR) —kg·m^2^** | 22.41 (20.20-24.77) | 21.49 (19.72-23.88) | 0.099 | 22.31 (20.31-24.32) | 22.71 (21.19-24.69) | 0.185 |
| **Infertile years (IQR) — yr** | 4.00 (3.00-6.00) | 3.00 (2.00-5.00) | 0.005 | 7.00 (3.00-11.00) | 5.50 (2.00-10.00) | 0.029 |
| Gravidity —no./total no. (%) |  |  | 0.048 |  |  | 0.345 |
| 0 | 370/553 (66.91) | 216/288 (75.00) |  | 192/373 (51.47) | 44/77 (57.14) |  |
| 1 | 117/553 (21.16) | 53/288 (18.40) |  | 75/373 (20.11) | 17/77 (22.08) |  |
| 2 | 43/553 (7.78) | 15/288 (5.21) |  | 52/373 (13.94) | 7/77 (9.09) |  |
| ≥3 | 23/553 (4.16) | 4/288 (1.39) |  | 54/373 (14.48) | 9/77 (11.69) |  |
| **FSH(IQR) —mIU/mL** | 6.74 (5.57-8.20) | 6.51 (5.21-7.72) | <0.001 | 7.55 (6.12-9.23) | 6.92 (5.46-8.03) | 0.038 |
| **E2(IQR) —pg/mL** | 144.00 (112.00-188.00) | 159.00 (121.75-211.75) | 0.367 | 153.50 (116.75-208.75) | 159.50 (120.00-209.00) | 0.618 |
| **PRL(IQR) —nmol/L** | 12.50 (9.10-18.30) | 11.95 (9.38-16.78) | 0.456 | 11.95 (8.52-16.55) | 12.40 (8.06-17.88) | 0.929 |
| **LH(IQR) —mIU/mL** | 3.97 (2.59-5.36) | 3.50 (2.42-5.04) | 0.536 | 3.52 (2.50-4.97) | 3.70 (2.85-4.68) | 0.810 |
| **Gonadotropin dose(IQR)—IU** | 2400.00 (1750.00-3225.00) | 2100.00 (1593.75-3000.00) | 0.003 | 3806.25 (2775.00-5013.75) | 2625.00 (2109.38-3468.75) | <0.001 |
| **Number of oocytes retrieval(IQR)** | 12.00 (8.00-17.00) | 13.50 (9.75-20.00) | <0.001 | 8.00 (5.00-12.00) | 11.00 (9.00-14.75) | <0.001 |
| **Number of oocytes used for r-ICSI** | 10.00 (6.00-13.00) | 10.00 (7.00-16.00) | 0.010 | 6.00 (4.00-10.00) | 9.00 (6.00-12.00) | <0.001 |
| **r-ICSI 2PN fertilization** | 0.50 (0.25-0.69) | 0.56 (0.29-0.74) | 0.087 | 0.50 (0.25-0.74) | 0.60 (0.37-0.77) | 0.001 |
| **IR—no./total no. (%)** | 55/1109 (4.96) | 149/338 (44.08) | <0.001 | 21/862 (2.44) | 35/95 (36.84) | <0.001 |
| **Positive hCG—no./total no. (%)** | 67/553 (12.12) | 164/288 (56.94) | <0.001 | 34/373 (9.11) | 36/77 (46.75) | <0.001 |
| **Clinical pregnancy—no./total no. (%)** | 45/553 (8.14) | 141/288 (48.96) | <0.001 | 16/373 (4.29) | 32/77 (41.55) | <0.001 |
| **MR—no./total no. (%)** | 12/45 (26.67) | 29/141 (20.57) | 0.390 | 6/16 (37.5) | 7/32 (21.88) | 0.310 |
| **LBR—no./total no. (%)** | 33/553(5.97) | 112/288 (38.89) | <0.001 | 10/373(2.68) | 25/77 (32.47) | <0.001 |
| **Newborns (N)** | 39 | 120 | - | 11 | 26 | - |
| **Cumulative clinical pregnancy—no./total no. (%)** | 56/553 (10.13) | 155/288 (53.82) | <0.001 | 20/373 (5.36) | 37/77 (48.05) | <0.001 |
| **cLBR—no./total no. (%)** | 51/553 (9.22) | 141/288 (48.96) | <0.001 | 16/373 (4.29) | 32/77 (41.56) | <0.001 |

*Note:* IR=implantation rate; MR=miscarriage rate; LBR=live birth rate; cLBR=cumulative live birth rate.

# Supplemental table 2.

**Supplemental table 2.** Comparison of the basic demographics and obstetric outcomes between the two age-adjusted cohort.

|  | **Fresh embryo transfer**  **(N=364)** | **Freeze-all-embryos(N=364)** | **P** |
| --- | --- | --- | --- |
| **Maternal age at oocyte retrieval (IQR) — yr** | 31.00 (29.00-34.00) | 31.00 (29.00-34.00) | 0.738 |
| **Maternal BMI (IQR) —kg·m^2^** | 22.27 (20.31-24.44) | 22.00 (20.20-24.02) | 0.256 |
| **Infertile years (IQR) — yr** | 5.00 (3.00-7.00) | 4.00 (2.00-6.00) | <0.001 |
| **FSH(IQR) —mIU/mL** | 7.00 (5.58-8.34) | 6.09 (4.63-7.71) | <0.001 |
| **E2(IQR) —pg/mL** | 141.50 (112.75-183.50) | 148.00 (105.25-200.00) | 0.435 |
| **PRL(IQR) —nmol/L** | 12.60 (9.14-18.70) | 12.10 (9.20-17.20) | 0.250 |
| **LH(IQR) —mIU/mL** | 3.66 (2.47-5.10) | 3.51 (2.07-5.05) | 0.218 |
| **Gonadotropin dose (IQR)—IU** | 2362.50 (1725.00-3287.50) | 2250.00 (1650.00-3075.00) | 0.065 |
| **Number of oocytes retrieval (IQR)** | 12.00 (7.25-17.00) | 13.00 (9.00-18.00) | <0.001 |
| **Number of oocytes used for r-ICSI** | 10.00 (6.00-14.00) | 10.00 (7.00-14.00) | 0.149 |
| **r-ICSI 2PN fertilization** | 0.50 (0.25-0.67) | 0.54 (0.33-0.72) | 0.009 |
| **Positive hCG—no./total no. (%)** | 78/364 (31.36) | 200/364 (54.94) | <0.001 |
| **Clinical pregnancy—no./total no. (%)** | 61/364 (16.76) | 173/364 (47.53) | <0.001 |
| **IR—no./total no. (%)** | 76/765 (9.93) | 184/432 (42.59) | <0.001 |
| **MR—no./total no. (%)** | 18/61 (29.51) | 36/173 (20.80) | 0.166 |
| **LBR—no./total no. (%)** | 43/364 (11.81) | 137/364 (37.64) | <0.001 |
| **Newborns (N)** | 50 | 146 | - |
| **Cumulative clinical pregnancy—no./total no. (%)** | 61/364 (16.76) | 192/364 (52.75) | <0.001 |
| **cLBR—no./total no. (%)** | 43/364 (11.81) | 160/364 (43.96) | <0.001 |

*Note:* IR=implantation rate; MR=miscarriage rate; LBR=live birth rate; cLBR=cumulative live birth rate.

# Supplemental table 3.

**Supplemental table 3.** Comparisons between intention-to-treat women undergoing fresh or freeze-all strategy in r-ICSI cycles.

|  | **Group 1** | **Group 2 & Group 3** | **P** |
| --- | --- | --- | --- |
|  | **Fresh embryo transfer (N=926)** | **Freeze-all-embryos combined with failed blastocyst culture (N=1081)** |  |
| **Maternal age at oocyte retrieval (IQR) — yr** | 33.00 (30.00-37.00) | 32.00 (29.00-36.00) | <0.001 |
| **Maternal BMI (IQR) —kg·m^2^** | 22.31 (20.20-24.61) | 22.10 (20.20-24.77) | 0.954 |
| **Infertile years (IQR) — yr** | 5.00 (3.00-8.00) | 4.00 (2.00-6.00) | <0.001 |
| **FSH (IQR) —mIU/mL** | 7.07 (5.60-8.64) | 7.05 (5.48-9.18) | <0.001 |
| **E2 (IQR) —pg/mL** | 145.00 (108.50-194.00) | 148.00 (107.00-195.50) | 0.128 |
| **PRL (IQR) —nmol/L** | 12.30 (8.86-17.60) | 11.70 (7.98-15.80) | 0.190 |
| **LH (IQR) —mIU/mL** | 3.40 (2.30-4.90) | 3.39 (2.10-5.10) | 0.001 |
| **Gonadotropin dose (IQR)—IU** | 2850.00 (1950.00-4015.63) | 2550.00 (1800.00-3600.00) | <0.001 |
| **Number of oocytes retrieval (IQR)** | 10.00 (6.00-15.00) | 10.00 (7.00-15.00) | 0.001 |
| **Number of oocytes used for r-ICSI (IQR)** | 8.00 (5.00-12.00) | 8.00 (5.00-11.00) | 0.023 |
| **r-ICSI 2PN fertilization** | 0.50 (0.25-0.71) | 0.50 (0.29-0.75) | 0.039 |
| **Newborns (N)** | 50 | 146 | - |
| **Conservative cumulative clinical pregnancy—no./total no. (%)** | 76/926 (8.21) | 192/1081 (17.76) | <0.001 |
| **Conservative cLBR—no./total no. (%)** | 57/926 (6.16) | 158/1081 (14.62) | <0.001 |

*Note:* cLBR=cumulative live birth rate.

# Supplemental table 4.

**Supplemental table 4.** The maternal characteristics of women experienced freeze-all-embryos procedure and failed blastocyst culture.

|  | **Freeze-all-embryos (N=365)** | **Failed blastocyst culture (N=716)** | **P** |
| --- | --- | --- | --- |
| **Maternal age at oocyte retrieval (IQR) — yr** | 31.00 (29.00-34.00) | 33.00 (30.00-37.00) | <0.001 |
| **Maternal BMI (IQR) —kg·m^2^** | 22.00 (20.20-24.01) | 22.24 (20.30-25.00) | 0.100 |
| **Infertile years (IQR) — yr** | 4.00 (2.00-6.00) | 4.00 (2.00-7.00) | 0.316 |
| **FSH(IQR) —mIU/mL** | 6.10 (4.64-7.71) | 6.33 (4.79-7.99) | 0.144 |
| **E2(IQR) —pg/mL** | 148.00 (105.50-200.00) | 154.00 (111.00-206.00) | 0.252 |
| **PRL(IQR) —nmol/L** | 12.10 (9.20-17.20) | 11.40 (8.20-17.10) | 0.342 |
| **LH(IQR) —mIU/mL** | 3.52 (2.08-5.05) | 3.07 (1.81-4.51) | 0.008 |
| **Gonadotropin dose(IQR)—IU** | 2250.00 (1650.00-3150.00) | 2775.00 (1881.25-3750.00) | <0.001 |
| **Number of oocytes retrieval(IQR)** | 13.00 (9.00-18.00) | 9.00 (7.00-13.00) | <0.001 |
| **Number of oocytes used for r-ICSI** | 10.00 (7.00-14.00) | 7.00 (4.00-10.00) | <0.001 |
| **r-ICSI 2PN fertilization** | 0.54 (0.33-0.72) | 0.50 (0.25-0.75) | 0.338 |

# Supplemental table 5.

**Supplemental table 5.** Demographic characteristics and clinical outcomes of participants with advanced age after age-stratified analysis.

|  | **≥35 AND <40 years (N=345)** | | | **≥40 AND ≤46 years(N=105)** | | |
| --- | --- | --- | --- | --- | --- | --- |
|  | **Fresh embryo transfer**  **(N=279)** | **Freeze-all-embryos(N=66)** | **P** | **Fresh embryo transfer**  **(N=94)** | **Freeze-all-embryos(N=11)** | **P** |
| **Maternal age at oocyte retrieval (IQR) — yr** | 37.00 (36.00-38.00) | 36.00 (35.00-37.00) | 0.010 | 41.00 (40.00-42.25) | 40.00 (40.00-41.00) | 0.174 |
| **Maternal BMI (IQR) —kg·m^2^** | 22.03 (20.18-23.69) | 22.58 (21.02-24.55) | 0.069 | 22.31 (20.31-24.32) | 22.67 (21.43-25.38) | 0.987 |
| **Infertile years (IQR) — yr** | 6.00 (3.00-10.00) | 5.00 (2.00-9.00) | 0.034 | 7.00 (2.00-14.00) | 5.00 (2.00-13.00) | 0.667 |
| **Gravidity —no./total no. (%)** |  |  | 0.708 |  |  | 0.742 |
| **0** | 146/279 (52.33) | 38/66 (57.58) |  | 46/94 (48.94) | 6/11 (54.55) |  |
| **1** | 58/279 (20.79) | 14/66 (21.21) |  | 17/94 (18.090 | 3/11 (27.27) |  |
| **2** | 40/279 (14.34) | 6/66 (9.09) |  | 12/94 （12.77） | 1/11 (9.09) |  |
| **≥3** | 35/279 (12.54) | 8/66 (12.12) |  | 19/94 (20.21) | 1/11 (9.09) |  |
| **FSH(IQR) —mIU/mL** | 7.48 (5.89-9.06) | 6.92 (5.15-8.18) | 0.073 | 8.10 (6.14-9.74) | 6.87 (5.31-10.21) | 0.551 |
| **E2(IQR) —pg/mL** | 149.00 (109.00-209.00) | 144.00 (106.00-199.00) | 0.469 | 149.50 (110.50-191.50) | 198.00 (91065-220.50) | 0.784 |
| **PRL(IQR) —nmol/L** | 11.65 (8.18-16.70) | 12.20 (8.11-18.30) | 0.799 | 11.32 (8.58-15.63) | 11.80 (3.10-14.10) | 0.562 |
| **LH(IQR) —mIU/mL** | 3.50 (2.39-5.04) | 3.70 (2.18-4.63) | 0.891 | 3.52 (2.50-4.97) | 3.70 (2.85-4.68) | 0.645 |
| **Gonadotropin dose(IQR)—IU** | 3675.00 (2625.00-4662.50) | 2550.00 (2065.50-3462.50) | <0.001 | 4287.50 (3018.75-5193.75) | 2775.00 (2531.25-3618.75) | 0.035 |
| **Number of oocytes retrieval(IQR)** | 8.00 (5.00-12.00) | 11.00 (8.00-14.00) | <0.001 | 7.00 (4.00-10.00) | 10.00 (8.00-14.00) | 0.013 |
| **Number of oocytes used for r-ICSI** | 7.00 (4.00-10.00) | 9.00 (6.00-11.25) | 0.006 | 6.00 (4.00-8.00) | 9.00 (5.00-10.00) | 0.083 |
| **r-ICSI 2PN fertilization** | 0.50 (0.27-0.75) | 0.57 (0.33-0.73) | 0.244 | 0.50 (0.18-0.72) | 0.60 (0.31-0.83) | 0.175 |
| **IR—no./total no. (%)** | 19/558 (3.41) | 30/80 (37.50) | <0.001 | 2/188 (1.06) | 5/15 (33.33) | <0.001 |
| **Positive hCG—no./total no. (%)** | 15/279 (5.38) | 31/66 (46.97) | <0.001 | 4/94 (4.26) | 5/11 (45.45) | <0.001 |
| **Clinical pregnancy—no./total no. (%)** | 15/279 (5.38) | 28/66 (42.42) | <0.001 | 1/94 (1.06) | 4/11 (36.36) | <0.001 |
| **MR—no./total no. (%)** | 6/15 (40.00) | 7/28 (25.00) | 0.307 | 0 | 0 | - |
| **LBR—no./total no. (%)** | 9/279 (3.23) | 21/66 (31.82) | <0.001 | 1/94 (1.06) | 4/11 (36.36) | <0.001 |
| **Newborns (N)** | 10 | 22 | - | 1 | 4 | - |
| **Cumulative clinical pregnancy—no./total no. (%)** | 18/279 (6.45) | 35/66 (53.03) | <0.001 | 2/94 (2.13) | 4/11 (36.36) | <0.001 |
| **cLBR—no./total no. (%)** | 12/279 (4.30) | 27/66 (40.91) | <0.001 | 2/94 (2.13) | 4/11 (36.36) | <0.001 |
